# The Influence of Body Composition on Sagittal Plane Posture among Elementary School-Aged Children

**DOI:** 10.3390/children11010036

**Published:** 2023-12-28

**Authors:** Bojan M. Jorgić, Stefan N. Đorđević, Miljan M. Hadžović, Saša Milenković, Nenad Đ. Stojiljković, Mihai Olanescu, Miruna Peris, Adrian Suciu, Danut Popa, Alin Plesa

**Affiliations:** 1 Faculty of Sport and Physical Education, University of Niš, 18000 Niš, Serbia; bojan.jorgic@fsfv.ni.ac.rs (B.M.J.); stefan.djordjevic@fsfv.ni.ac.rs (S.N.Đ.); miljan.hadzovic@fsfv.ni.ac.rs (M.M.H.); sasa.milenkovic@fsfv.ni.ac.rs (S.M.); nenad@fsfv.ni.ac.rs (N.Đ.S.); 2 Faculty of Automotive, Mechatronics and Mechanical Engineering, Technical University of Cluj-Napoca, 400641 Cluj-Napoca, Romania; adrian.suciu@mdm.utcluj.ro (A.S.); ilie.popa@mdm.utcluj.ro (D.P.); alin.plesa@mdm.utcluj.ro (A.P.); 3 Faculty Industrial Engineering, Robotics and Production Management, Technical University of Cluj-Napoca, 400641 Cluj-Napoca, Romania; miruna.peris@muri.utcluj.ro

**Keywords:** body posture, fat mass, muscle mass, spine, children

## Abstract

Proper posture, characterized by the appropriate alignment of the cervical, thoracic, and lumbar segments of the spine, enables these regions to maintain their normal curvature. Body composition is recognized as one of the factors that can influence overall postural alignment of the spine. The objective of this study was to determine the influence of the parameters of body composition on the prevalence of postural disorders in the sagittal plane. The cross-sectional study was conducted on 152 children of both genders (78 boys), at a mean age of 11 years ± 6 months. In order to evaluate postural disorders, the Formetric 4D System, a tool manufactured by Diers, Schlangenbad, Germany was used. Based on its output data, the following variables were obtained: hyperkyphosis, hyperlordosis, kypholordosis, flatback, and normal alignment of the body in the sagittal plane. The evaluation of body composition parameters was conducted using the InBody 770 device. To determine how body composition influences the postural status of the spinal column, a discriminant analysis was employed. The results showed that approximately 65.8% of children exhibit various types of postural disorders when assessing the alignment of the spine in the sagittal plane. The most prevalent disorder observed was hyperkyphosis, affecting 34.2% of the subjects, followed by kypholordosis at 16.4%. Moreover, the results demonstrated that body composition significantly influences body posture (*p* = 0.004). An increase in fat mass corresponds to a deviation from normal body posture, whereas an increase in the percentage of skeletal muscle mass and fat-free mass is associated with a reduction in postural abnormalities in the sagittal plane. Considering the results, it is clear that body composition parameters serve as more reliable predictors of the influence on body posture compared to simply calculating the body mass index. Furthermore, it can be concluded that there are consistent patterns of influence by specific body composition parameters, including fat mass, percentage of skeletal muscle, and fat-free mass, on body posture among children from various climates. These results underscore the significance of implementing strength exercises in children, particularly during periods of rapid growth and development, as a means of preventing and correcting postural disorders.

## 1. Introduction

Body posture is primarily a dynamic stereotype shaped by both conditional and unconditional reflexes [1]. Maintaining proper posture, which involves the correct alignment of the spinal column’s cervical, thoracic, and lumbar curvatures within established normal values, is commonly assessed using angles [2]. Any deviation, be it an increase or decrease in these spinal curvature angles, can result in improper body posture and the development of postural deformities. Some of the most common deformities in the sagittal plane are hyperkyphosis and hyperlordosis, followed by flatback and kypholordosis [3,4]. Postural issues are prevalent among children aged 3 to 18 and constitute a widespread global health concern [5]. The existing literature suggests that long-term exposure to poor postural habits associated with various aspects of modern lifestyles contributes to postural deviations [6]. Factors such as gender, race, mental well-being, locomotor system structure, physical inactivity, sedentary living, unhealthy dietary habits, and more have been identified as common culprits [7]. Furthermore, as children undergo growth and development, their body posture can undergo changes influenced by morphological attributes and body composition [8,9,10]. Research studies have indicated a higher incidence of postural disorders among overweight and obese children, establishing a link between nutritional level, body mass index, and postural status [11,12,13].

Sometimes, a child may have elevated body fat content, irrespective of whether their body mass index (BMI) falls within normal ranges [14]. To gain more precise insights, it is crucial to pinpoint which body composition parameters influence body posture. This approach allows us to discern the positive or negative influences of fat mass, muscle mass, and fat-free mass on body posture. Furthermore, it helps identify which body composition parameters play a more significant role in particular postural disorders like hyperkyphosis, hyperlordosis, scoliosis, and others. Previous research has already established varying associations between different body composition parameters and specific postural disorders. For instance, one study linked body fat mass to lumbar lordosis and the inclination of the body’s long axis [15]. Araujo et al. found that both body fat mass and fat-free mass correlate with the lumbar angle, and this held true for both boys and girls [16]. Few studies have delved into the connection between skeletal muscle mass and postural status [1]. Exploring the link between skeletal muscle mass and postural status can help emphasize the significance of physical activity among children, particularly during phases of rapid growth and development when many postural disorders manifest. Earlier research suggests that these disorders may stem from variations in the rates of bone and muscle growth, often influenced by neuro-hormonal changes during puberty [2,17].

Building upon the points discussed earlier, the objective of this study was to determine the influence of the parameters of body composition on the prevalence of postural disorders in the sagittal plane.

## 2. Materials and Methods

### 2.1. Subjects

The participant sample comprised 152 fifth-grade children, averaging 11 years with a variation of ±6 months in age, who attended elementary schools in Knjaževac, Serbia. Out of the entire sample, 78 were boys, while 74 were girls. The sample of 152 children consisted of more than 90% of fifth-grade children. Written parental consent was obtained for every child participating in the research. The criteria for inclusion in the study required that the children did not have any diseases or disorders that could lead to postural deformities, such as muscular dystrophy, cerebral palsy, spina bifida, and similar conditions. Furthermore, the inclusion criterion was that the respondents did not have structural scoliosis, which, due to its shape, could lead to an influence on the shape of the anterior-posterior curvatures of the spine. The participants underwent testing within the framework of the project “Development of diagnostic centers for postural and musculoskeletal disorders in school children in Serbia and Bulgaria” implemented by the Sports Association of the City of Knjaževac, Serbia.

### 2.2. The Sample of Variables

#### 2.2.1. Variables for the Evaluation of the Postural Status in the Sagittal Plane

The assessment of postural disorders was conducted using the Formetric 4D System (Diers, Schlangenbad, Germany), a non-invasive diagnostic tool that employs photometry [18,19,20]. Previous research has confirmed its validity and reliability [21,22]. The following postural variables were evaluated utilizing the Formetric 4D System instrument: hyperkyphosis (HKYP), hyperlordosis (HLOR), kypholordosis (KYPLOR), flatback (FB), and normal body posture (NBP).

A description of the isolated variables is given in Table 1. The variables are described based on the output information and the angles provided by the Formetric 4D System instrument. All variables for the assessment of body posture are defined according to the American Scoliosis Research Society, Wenger et al. [23], and Shefi et al. [24].

#### 2.2.2. Variables for Appraising Body Composition

Body composition variables were conducted using the InBody 770 instrument (InBody Co., Seoul, Republic of Korea), which has been validated and proven reliable for use among children in previous research [25,26]. The following variables of body composition were used: body fat mass in kg (BFM), percent body fat in % (PBF), skeletal muscle mass in kg (SMM), percent skeletal muscle mass in % (PSMM), fat-free mass in kg (FFM), and percent fat-free mass in % (PFFM). The variable for the percent skeletal muscle mass of the body (PSMM) was calculated as follows: the value of skeletal muscle mass of the body in kilograms (SMM) was divided by the value of body mass in kilograms and then multiplied by 100. Similarly, the variable for percent fat-free mass in % (PFFM) was calculated by dividing the obtained value of fat-free mass in kilograms by the value of body mass in kilograms and then multiplying by 100.

### 2.3. Research Protocol

A cross-sectional study was employed to determine the influence of body composition parameters on the prevalence of postural disorders in the sagittal plane. The testing for postural disorders and body composition was conducted in the diagnostic center of the Sports Association of Knjaževac. The assessments were conducted in the morning, between 8 am and 11 am, following standardized testing procedures. Trained examiners, authorized to operate the ‘DIERS’ 4D photometric instrument (Formetric 4D System, Diers, Schlangenbad, Germany), performed the postural assessments. These assessments were conducted in a partially lit room, with participants wearing minimal clothing. Markers were placed on specific anatomical points, such as the fossae lumbales laterales and C7-processus spinosus. Subsequently, participants were positioned on the instrument’s platform with their feet parallel and their backs facing the instrument’s camera. The instrument’s software was utilized to calculate and report the results. Body composition on the InBody 770 instrument was measured based on a standard protocol which is used among children [27]. Considering that the participants were underaged children, the guidelines for testing body composition were given to their parents. Preparations before the testing included refraining from physical activity 24 h before the test and avoiding caffeine or any other fluid or food consumption 12 h before the testing. Compliance with these recommendations was assessed using the InBody 770 instrument prior to testing. During the actual testing, children were required to be barefoot and wear minimal clothing.

Approval for all procedures in this study was granted by the Ethics Committee of the Faculty of Sport and Physical Education in Niš (reference number 04-542/2), and the research was conducted in adherence to the principles articulated in the Declaration of Helsinki [28].

### 2.4. Statistical Analysis

Variables related to postural status were expressed as frequencies (numbers) and percentages (%). For variables related to body composition, basic descriptive statistics parameters were calculated, including arithmetic means (Mean) and standard deviation (SD). Given the fact that the results were presented on two distinct numerical scales, nominal for postural disorders and rational for body composition, a discriminant analysis was employed to assess the influence of body composition on the postural status in the sagittal plane. Discriminant analysis allows for the creation of models to predict and differentiate respondents into specific categories. This analysis provides an assessment of the influence of each variable on this differentiation, indicating the presence of differences between the observed categories. A significance level for statistical analysis was set at *p* < 0.05. The data were analyzed using IBM SPSS Statistics for Windows, Version 19.0, developed by IBM Corp. (Armonk, NY, USA) in 2010.

## 3. Results

The outcomes presented in Table 2 reveal that, among all the children, 65.8% exhibit postural disorders in the sagittal plane (PDSP). When looking at individual disorders, hyperkyphosis (HKYP) is the most common at 32.2%, while hyperlordosis (HLOR) is the least common at 4.6%. Among male participants, 69.2% exhibit postural disorders in the sagittal plane, and 62.2% of the female participants exhibit these disorders. For males, hyperkyphosis (HKYP) is the most prevalent at 35.9%, while hyperlordosis (HLOR) is the least prevalent at 3.8%. Among the female participants, hyperkyphosis (HKYP) is the most common at 28.4%, and hyperlordosis (HLOR) is the least common at 5.4%. 

The outcomes presented in Table 3 indicate that there are differences in skeletal muscle mass, both in kilograms and as a percentage, between male and female participants (16.93 kg vs. 16.25 kg; 40.38% vs. 38.11%). Additionally, there are variations in other parameters of body composition.

The outcomes in Table 4 indicate a statistically significant (*p* = 0.004) contribution or influence of body composition on the postural status of the spinal column in the sagittal plane for the entire participant sample. Notably, parameters such as the percent of muscle mass (−0.626) and the percent of fat-free mass (−0.531) have negative values, suggesting an inverse relationship with postural status. The numeric values for the remaining parameters, indicating their influence on the postural status, have positive signs.

## 4. Discussion

The data concerning postural status (Table 1) reveal that more than half of the fifth-grade children, specifically 65.8%, exhibit deviations from normal body posture in the sagittal plane. These findings are consistent with previous studies involving participants from the same geographic area. Jorgić et al. [30] reported a prevalence of postural disorders greater than 50% among children from the first to the fourth grades. Among 515 subjects, 326 individuals were identified with various postural disorders in the sagittal plane, specifically including flatback, hyperkyphosis, hyperlordosis, and kypholordosis. In another study, the authors [31] also found high prevalence of postural disorders in the sagittal plane (60.4%) such as kypholordosis, flatback, hyperkyphosis, and hyperlordosis when working with eighth graders. Similar results were obtained in studies conducted in other countries. A study conducted in the Slovak Republic also indicated that over 50% of children have poor body posture [32]. A study involving 2129 children from Bulgaria, aged 6 to 9, found that 58.85% of them had postural disorders, with 23.67% having spinal deformities [33]. Analyzing various studies, the authors Batistão et al. [34] noted that over 50% of healthy children display various types of postural disorders, with hyperlordosis and hyperkyphosis being common. The observed percentage of HKYP varies between boys and girls (5.9% vs. 28.4%), which aligns with results from the study by Poussa et al. [35]. In their research involving participants aged 11 to 22, they found that boys in all age categories had a greater kyphosis angle compared to girls, while girls had higher values for the lordosis angle. A separate study [36] on participants aged 7 to 11 also reported an increased occurrence of HKYP among boys and heightened prevalence of HLOR among girls. The outcomes from our research also align with findings from a study involving children of both sexes, aged 8 to 16 years, conducted in Poland [37]. According to the results of the referenced study, boys aged 11 exhibit higher prevalence of thoracic kyphosis compared to girls.

An analysis of the influence of body composition on postural status revealed that all the measured body composition parameters had a significant effect (*p* = 0.004) on the development of postural disorders in the sagittal plane (Table 3). Specifically, when examining body fat parameters, both absolute and relative values (BFM and PBF) demonstrated a statistically significant negative influence on the occurrence of postural deformities in the sagittal plane (0.732 and 0.750). This implies that as body fat increases, the likelihood of postural abnormalities in the anteroposterior orientation and deviations from normal body posture also increases. A study by Araujo et al. [16] identified a statistically significant association between body composition parameters and postural status in the sagittal plane, showing a positive association between fat, fat-free mass, and the lumbar angle (*p* < 0.001). Additionally, a more comprehensive study by Jankowicz-Szymańska et al. also indicated that body fat mass negatively affects the prevalence of alignment abnormalities in the anteroposterior orientation [15]. In their study involving 910 children aged 10–12, researchers found a correlation between body fat mass and lumbar lordosis, indicating that an augmentation in body mass is linked with an increase in lumbar lordosis. They also determined that overweight children have higher prevalence of hyperlordosis in contrast to children with average weight (53.7% vs. 43.8%). Grabara et al. [37] also identified an association between sagittal curvatures and body composition parameters, although the observed correlations were found to be modest. An additional study conducted on black South African children aged 11 to 13 in the North-West Province demonstrated a strong association between lordosis in boys and both BMI and the percentage of fat mass [38]. When studying the influence of body composition on postural status in the frontal plane (scoliosis) using the same sample of participants as in our study, Kurtović et al. determined that an increase in PBF and BFM leads to an increased likelihood of scoliosis development [39]. The impact of body fat on posture appears to be consistent regardless of the place of residence, geographical region, or race.

Skeletal muscle mass typically increases during rapid growth periods, especially during puberty and during adolescence [40]. When analyzing postural status, it is more meaningful to consider relative values, such as the percentage of skeletal muscle mass (PSMM). The obtained results suggest that PSMM has a positive influence on body posture in the sagittal plane (−0.626). The minus sign “−” indicates that an increase in PSMM in children aged approximately 11 is associated with a reduced likelihood of deviating from a typical body posture in the sagittal plane. In other words, it decreases the risk of developing postural disorders like hyperkyphosis or hyperlordosis, etc. The same positive influence on reducing the deviation from the typical bodily alignment in the anteroposterior orientation was observed for PFFM (−0.531). Golalizadeh et al. [41], in a study with somewhat older girls (14–18 years of age), found that fat-free mass had a protective effect against postural disorders. When analyzing the influence of body composition relative to gender, it is evident that, in both boys and girls, PSMM and PFFM exert a beneficial impact on reducing the likelihood of deviating from normal body posture in the sagittal plane. In a study similar to ours and involving participants aged 10–12, Wilczynski et al. [1] discovered that skeletal muscle mass, fat-free mass, and body fat mass are significant body composition parameters that influence differences in the postural status in the sagittal plane.

The findings indicate that an enhancement in muscle mass and strength in the entire body, particularly in the back and core stability muscles, can reduce the likelihood of hyperlordosis and hyperkyphosis occurring. Research in this field indicates that body posture relies on the proper and coordinated function of the muscles responsible for spinal movement and the even distribution of their strength [33]. Children with strong musculature, particularly mesomorphs, typically experience fewer postural disorders [1]. Strengthening the abdominal muscles has been shown to decrease the lumbar lordosis angle, as confirmed in multiple studies [42,43]. This is particularly important since an increase in the lordosis angle can potentially affect the positioning of the pelvis in the anteroposterior orientation, resulting in alterations in the hip joints and an increased risk of lower back pain [15]. By incorporating strength exercises for the back muscles, especially in the thoracic part of the spine, it is possible to prevent and correct an increase in the curvature of the mid-back spine, thus preventing hyperkyphosis. The favorable impact of corrective exercises on hyperkyphosis among 11-to-12-year-old boys was supported by the research carried out by Rajabi et al. [44]. Furthermore, the beneficial effects of strength exercises on the correction of hyperkyphosis were affirmed in older participants at an average age of 21.95 ± 3.90 years, according to the study by Park et al. [45].

The results suggest that increasing skeletal muscle mass through physical exercise and participation in various physical activities is crucial. Research by Wyszyńska et al. [46] confirmed that the extent of physical exertion, combined with body composition, influences variations in postural status parameters among 11-to-13-year-old participants. Balkó et al. [47] found that poor posture was prevalent in 83% of 10-to-11-year-old children who did not engage in sports or were only physically active once a week. Conversely, children who were physically active for more than 3 h a week tended to have better posture (*p* = 0.0001). The study by Molina-Garcia et al. [48] found that various components of physical fitness and functional movement collectively contribute to improved postural status and better body posture among children of a specific age (10.86 ± 1.25 years), especially those dealing with obesity or overweight. When examining these components individually, the researchers observed that children who performed better in tests related to 1RM arm and handgrip strength, cardiorespiratory fitness, and agility tended to exhibit more aligned lumbar and thoracic spine posture. The findings from this study, along with those from our research and other similar studies, underscore the importance of regular physical exercise for children who do not have postural disorders. This aligns with the exercise recommendations provided by the American College of Sports Medicine [49,50,51]. For children who exhibit deviations from normal body posture, it is essential to implement exercise programs aimed at correcting these postural disorders, in addition to promoting recommended physical activity.

This study exhibits several strengths. It demonstrates consistent patterns of influence from specific body composition parameters on body posture in children from various climates. Given that an increase in the percentage of skeletal muscle mass contributes to better posture, we assume, following the results of other studies that we have mentioned, which deal with the effects of core stability exercise on postural status, that it is very important to incorporate strength exercises, particularly those targeting the muscles responsible for core stability, in preventing and correcting postural issues.

However, this study does have certain limitations that require consideration. The application of bioelectrical impedance analysis (BIA) for evaluating body composition, while valid and reliable among children, may have limitations, particularly in cases of obese children. It is worth noting that there might be less congruence between BIA results and the standard dual-energy X-ray absorptiometry (DXA) in children with mild-to-moderate obesity [52]. To minimize this discrepancy, the study employed one of the latest BIA devices, the InBody 770, which uses Tetrapolar 8-Point Tactile Electrodes. Furthermore, one of the limitations of the conducted study is that only 11-year-old children were included in the research. In order to obtain general conclusions about the influence of body composition on body posture, research should be conducted on a sample of children of different age groups. Additionally, a potential avenue for further research involves dividing participants into subgroups based on their BMI, which could provide insights into how body composition influences body posture concerning nutritional status. 

## 5. Conclusions

The study found that a significant number of children, specifically 65.8%, exhibit various types of postural disorders in the sagittal plane of the spinal column. Furthermore, it identified a statistically significant relationship between body composition and deviations from a normal posture. Increased body fat mass is linked to the development of poor posture, while an increase in the percentage of muscle mass and the percentage of fat-free mass is associated with a decrease in alignment irregularities in the anteroposterior orientation of the spinal column. To address and prevent these issues, based on the results of our study and additional analyzed research it is essential to focus on strengthening core body muscles and all the back muscles in children, particularly during periods of rapid growth and development. 

## Figures and Tables

**Table 1:** A description of the variables utilized to evaluate postural status in the sagittal plane.

Variable	Description
Hyperkyphosis (HKYP)	Hyperkyphosis is a curvature in the thoracic part of the spinal column in the sagittal plane, with an angle greater than 45°.
Hyperlordosis (HLOR)	Hyperlordosis is a curvature in the lumbar part of the spinal column in the sagittal plane, with an angle greater than 48° among children aged 11 to 13.
Kypholordosis (KYPLOR)	Kypholordosis is a postural disorder characterized by the simultaneous presence of an excessive curvature in the thoracic part of the spinal column (greater than 45°) and an excessive curvature in the lumbar part of the spinal column (greater than 48°). This condition is typically observed among children aged 11 to 13.
Flatback (FB)	Flatback is a postural disorder characterized by reduced angles of the normal physiological curvatures in the spine. Among participants aged 11 to 13, it typically involves an angle of less than 20° in the thoracic part and less than 30° in the lumbar part of the spine.
Normal body posture in the sagittal plane (NBP)	Normal body posture is characterized by the presence of normal physiological curvatures in the spine. In children aged 11 to 13, the normal curvature range is typically between 20° and 45° in the thoracic part of the spine and between 30° and 48° in the lumbar part of the spine.

**Table 2:** Spinal column postural disorders in the sagittal plane.

Variable		NBP	HKYP	HLOR	KYPLOR	FB	PDSP
G	N	(N/%)	(N/%)	(N/%)	(N/%)	(N/%)	(F/%)
B	78	24/30.8%	28/35.9%	3/3.8%	9/11.5%	14/17.9%	54/69.2%
G	74	28/37.8%	21/28.4%	4/5.4%	16/21.4%	5/6.8%	46/62.2%
T	152	52/34.2%	49/32.2%	7/4.6%	25/16.4%	19/12.5%	100/65.8%

Legend: G—gender, N—number, B—boys, G—girls, T—total, HKYP—hyperkyphosis, HLOR—hyperlordosis, KYPLOR—kypholordosis, FB—flatback, NBP—normal body posture in the sagittal plane, PDSP—total postural disorders in the sagittal plane.

**Table 3:** Parameters of body composition.

Variable		SMM		PSMM		BFM		PBF		FFM		PFFM	
G	N	Mean	SD	Mean	SD	Mean	SD	Mean	SD	Mean	SD	Mean	SD
M	78	16.93	3.09	40.38	5.46	11.22	9.06	23.03	11.07	25.69	5.38	60.79	7.07
F	74	16.25	3.40	38.11	4.96	12.63	7.97	26.73	10.11	24.60	5.59	57.33	6.34
T	152	16.59	3.24	39.27	5.33	11.91	8.54	24.82	10.74	25.16	5.49	59.11	6.92

Legend: G—gender, N—number, M—male, F—female, T—total subjects, Mean—arithmetic means, SD—standard deviation. BFM—body fat mass in kg, PBF—percent body fat in %, SMM—skeletal muscle mass in kg, PSMM—percent muscle mass in %, FFM—fat-free mass in kg, and PFFM—percent fat-free mass in %.

**Table 4:** The influence of body composition parameters on postural status in the sagittal plane. Adapted from Đorđević [29].

G	SMM	PSMM	BFM	PBF	FFM	PFFM	*p*	WL	Cs	CC	Ei	%CC
B	0.681	−0.772	0.829	0.875	0.723	−0.688	0.062	0.848	12.007	0.389	0.179	57.7
G	0.347	−0.435	0.485	0.534	0.380	−0.360	0.109	0.878	9.010	0.349	0.138	66.2
T	0.593	−0.626	0.732	0.750	0.640	−0.531	**0.004**	0.889	17.358	0.333	0.125	68.4

Legend: G—gender, B—boys, G—girls, T—total, BFM—body fat mass in kg, PBF—percent body fat in %, SMM—skeletal muscle mass in kg, PSMM—percent muscle mass in %, FFM—fat-free mass in kg, and PFFM—percent fat-free mass in %, WL—Wilks’s lambda-level of discriminatory power; *p*—level of statistical significance; Cs—Chi-square significance of the connection of the researched areas; CC—canonical correlation; Ei—eigenvalues; %CC—grouped cases correctly classified. Bold means statistical saginificant.

## Data Availability

Data available on request due to restrictions, e.g., privacy or ethics. Data presented in this study are available upon request from the corresponding author. The data are not publicly available to protect the privacy of the participants in this research.

## References

[1] Wilczyński J., Lipińska-Stańczak M., Wilczyński I. (2020). Body posture defects and body composition in school-age children. Children.

[2] Živković D. (2009). Osnove Kineziologije sa Elementima Kliničke Kineziologije [An Introduction to Kinesiology with Elements of Clinical Kinesiology].

[3] Bullock-Saxton J. (1988). Normal and abnormal postures in the sagittal plane and their relationship to low back pain. Physiother. Pract..

[4] Czaprowski D., Stoliński Ł., Tyrakowski M., Kozinoga M., Kotwicki T. (2018). Non-structural misalignments of body posture in the sagittal plane. Scoliosis Spinal Disord..

[5] Rusek W., Baran J., Leszczak J., Adamczyk M., Weres A., Baran R., Inglot G., Pop T. (2018). The influence of body mass composition on the postural characterization of school-age children and adolescents. BioMed Res. Int..

[6] Quka N., Stratoberdha D.H., Selenica R. (2015). Risk factors of poor posture in children and its prevalence. Acad. J. Interdiscip. Stud..

[7] Latalski M., Bylina J., Fatyga M., Repko M., Filipovic M., Jarosz M.J., Borowicz K.B., Matuszewski Ł., Trzpis T. (2013). Risk factors of postural defects in children at school age. Ann. Agric. Environ. Med..

[8] Gilleard W., Smith T. (2007). Effect of obesity on posture and hip joint moments during a standing task, and trunk forward flexion motion. Int. J. Obes..

[9] Smith A.J., O’Sullivan P.B., Beales D.J., De Klerk N., Straker L.M. (2011). Trajectories of childhood body mass index are associated with adolescent sagittal standing posture. Int. J. Pediatr. Obes..

[10] Araújo F., Lucas R. (2014). What do we know about the determinants of sagittal standing posture?. OA Musculoskelet. Med..

[11] Protić-Gava B., Šćepanović T., Batez M. (2015). The incidence of postural disorders with regard to degree of nutritional status in adolescents. Sport Mont.

[12] Lubkowska W., Mroczek B. (2017). Assessment of body posture of boys aged 7-15 in relation to the body mass index–BMI. J. Educ. Health. Sport.

[13] Grabara M., Pstrągowska D. (2008). Estimation of the body posture in girls and boys related to their body mass index (BMI). Pol. J. Sports Med..

[14] Guerrero-Romero F., Rodriguez-Moran M. (2012). Metabolically obese normal-weight children. World. J. Clin. Pediatr..

[15] Jankowicz-Szymańska A., Bibro M., Wodka K., Smola E. (2019). Does excessive body weight change the shape of the spine in children?. Child. Obes..

[16] Araujo F.A., Simoes D., Silva P., Alegrete N., Lucas R. (2019). Sagittal standing posture and relationships with anthropometrics and body composition during childhood. Gait Posture.

[17] Medojević S., Jakšić D. Razlike u posturalnim poremećajima između devojčica i dečaka od 7-15 godina na teritoriji Vojvodine [Differences in postural disorders between boys and girls aged 7 to 15 on the territory of Vojvodina]. Proceedings of the Interdisciplinary Scientific Conference “Anthropological Status and Physical Activity of Children, Youth and Adults”.

[18] Đorđević S., Vidojević M., Đokić M., Milenković M., Stanković R. The postural status of female first league basketball players. Proceedings of the 21st Scientific Conference, FIS Communications 2018” in Physical Education, Sport and Recreation.

[19] Betsch M., Wild M., Jungbluth P., Hakimi M., Windolf J., Haex B., Horstmann T., Rapp W. (2011). Reliability and validity of 4D rasterstereography under dynamic conditions. Comput. Biol. Med..

[20] Mangone M., Raimondi P., Paoloni M., Pellanera S., Di Michele A., Di Renzo S., Vanadia M., Dimaggio M., Murgia M., Santilli V. (2013). Vertebral rotation in adolescent idiopathic scoliosis calculated by radiograph and back surface analysis-based methods: Correlation between the Raimondi method and rasterstereography. Eur. Spine J..

[21] Somoskeöy S., Tunyogi-Csapó M., Bogyó C., Illés T. (2012). Clinical validation of coronal and sagittal spinal curve measurements based on three-dimensional vertebra vector parameters. Spine J..

[22] Lason G., Peeters L., Vandenberghe K., Byttebier G., Comhaire F. (2015). Reassessing the accuracy and reproducibility of Diers formetric measurements in healthy volunteers. Int. J. Osteopath. Med..

[23] Wenger D.R., Frick S.L. (1999). Scheuermann kyphosis. Spine.

[24] Shefi S., Soudack M., Konen E., Been E. (2013). Development of the lumbar lordotic curvature in children from age 2 to 20 years. Spine.

[25] Kriemler S., Puder J., Zahner L., Roth R., Braun-Fahrländer C., Bedogni G. (2008). Cross-validation of bioelectrical impedance analysis for the assessment of body composition in a representative sample of 6-to 13-year-old children. Eur. J. Clin. Nutr..

[26] Lim J.S., Hwang J.S., Lee J.A., Kim D.H., Park K.D., Jeong J.S., Cheon G.J. (2009). Cross-calibration of multi-frequency bioelectrical impedance analysis with eight-point tactile electrodes and dual-energy X-ray absorptiometry for assessment of body composition in healthy children aged 6–18 years. Pediatr. Int..

[27] Kutac P., Bunc V., Sigmund M. (2020). Determination of Body Fat Ratio Standards in Children at Early School Age Using Bioelectric Impedance. Medicina.

[28] World Medical Association (2011). Handbook of WMA Policies. http://www.wma.net/en/30publications/10policies/b3/index.html.

[29] Đorđević S. (2022). Postural Status and Body Composition of School-Age Children. Ph.D. Thesis.

[30] Jorgić B., Milenković M., Ždrele S., Milenković S., Stanković R., Bubanj S. (2015). Spinal cord posture in the sagittal plane among young schoolchildren residing in the area of Knjaževac. Facta. Univ. Ser. Phys. Educ. Sport.

[31] Jorgić B., Đorđević S., Milenković S., Stanković R. (2020). The prevalence of postural disorders among eighth grade elementary school students. Phys. Educ. Sport Through Centuries.

[32] Kolarová M., Kutiš P., Rusnák R., Hrčková Z., Hudáková Z., Lysá Ľ., Luliak M., Babeľa R. (2019). Analysis of body segments and postural state in school children. Neuro. Endocrinol. Lett..

[33] Mitova S. (2015). Frequency and prevalence of postural disorders and spinal deformities in children of primary school age. Res. Kinesiol..

[34] Batistão M.V., Moreira R.D.F.C., Coury H.J.C.G., Salasar L.E.B., Sato T.D.O. (2016). Prevalence of postural deviations and associated factors in children and adolescents: A cross-sectional study. Fisioter. Mov..

[35] Poussa M.S., Heliövaara M.M., Seitsamo J.T., Könönen M.H., Hurmerinta K.A., Nissinen M.J. (2005). Development of spinal posture in a cohort of children from the age of 11 to 22 years. Eur. Spine. J..

[36] Pokrywka J., Fugiel J., Posłuszny P. (2011). Prevelance of postural disorders in children from Copper Basin in Poland. Fizjoterapia.

[37] Grabara M., Bieniec A., Nawrocka A. (2017). Spinal curvatures of children and adolescents—A cross-sectional study. Biomed. Hum. Kinet..

[38] Stroebel S., De Ridder J.H., Wilders C.J., Ellis S.M. (2009). Influence of body composition on the prevalence of postural deformities in 11 to 13 year old black South African children in the North-West Province. S. Afr. J. Res. Sport. Ph..

[39] Kurtović N., Skender N., Čolakhodzić E., Mahmutović I., Kendić S., Porić S. (2021). Prevalence of spine deformities and relationship with body composition in eleven and twelve year old students. Sport. Logos.

[40] Brown K.A., Patel D.R., Darmawan D. (2017). Participation in sports in relation to adolescent growth and development. Transl. Pediatr..

[41] Golalizadeh D., Toopchizadeh V., Fasaie N., Dolatkhah N. (2019). Body composition indices in a sample of female adolescents with postural deformity: A case control study. BMC Res. Notes.

[42] Hosseinifar M., Ghiasi F., Akbari A., Ghorbani M. (2017). The effect of stabilization exercises on lumbar lordosis in patients with low back pain. Ann. Trop. Med. Public Health.

[43] Rahmani M., Zandi S., Minoonejad H. (2022). Core stability based corrective exercise program on improving and functional movement patterns in male adults with lumbar hyper-lordosis. Int. J. Musculoskelet. Pain Prev..

[44] Rajabi F., Minoonejad H., Seidi F., Rajabi R. (2022). To compare the effect and sustainability of a course corrective games with selected corrective exercise on 10-12 aged boys with hyper thoracic kyphosis. J. Paramed. Sci. Rehabil..

[45] Park Y.J., Kim W.M., Yu J.H., Moon H.H., Seo Y.G. (2022). Effects of combined exercise program on spinal curvature and balance ability in adolescents with kyphosis. Children.

[46] Wyszyńska J., Podgórska-Bednarz J., Drzał-Grabiec J., Rachwał M., Baran J., Czenczek-Lewandowska E., Leszczak J., Mazur A. (2016). Analysis of relationship between the body mass composition and physical activity with body posture in children. BioMed Res. Int..

[47] Balkó S., Balkó I., Valter L., Jelínek M. (2017). Influence of physical activities on the posture in 10–11 year old schoolchildren. J. Phys. Educ. Sport.

[48] Molina-Garcia P., Plaza-Florido A., Mora-Gonzalez J., Torres-Lopez L.V., Vanrenterghem J., Ortega F.B. (2020). Role of physical fitness and functional movement in the body posture of children with overweight/obesity. Gait Posture.

[49] American College of Sports Medicine (2013). ACSM’s Guidelines for Exercise Testing and Prescription.

[50] Mocanu G., Murariu G. (2023). The influence of the specificity of sports specializations on the values of muscle power for female university students. Balneo PRM Res. J..

[51] Mocanu G.D., Murariu G., Onu I. (2022). The Influence of BMI Levels on the Values of Static and Dynamic Balance for Students (Men) of the Faculty of Physical Education and Sports. J. Men’s Health.

[52] Seo Y.G., Kim J.H., Kim Y., Lim H., Ju Y.S., Kang M.J., Lee K., Lee H., Jang H.B., Park S.I. (2018). Validation of body composition using bioelectrical impedance analysis in children according to the degree of obesity. Scand. J. Med. Sci. Sports.

